# A pilot study of an exercise & cognitive behavioral therapy intervention for epithelial ovarian cancer patients

**DOI:** 10.1186/1757-2215-6-21

**Published:** 2013-04-04

**Authors:** Shalini H Moonsammy, Crissa L Guglietti, Daniel Santa Mina, Sarah Ferguson, Jennifer L Kuk, Sara Urowitz, David Wiljer, Paul Ritvo

**Affiliations:** 1 School of Kinesiology and Health Science, York University, 4700 Keele Street, Toronto, Ontario, M3J 1P3, Canada; 2 Kinesiology Program, University of Guelph-Humber, Toronto, Ontario, Canada; 3 Survivorship Exercise Program, University Health Network, Toronto, Ontario, Canada; 4 Division of Gynecologic Oncology, Princess Margaret Hospital, Toronto, Ontario, Canada; 5 Department of Obstetrics and Gynecology, University of Toronto, Toronto, Ontario, Canada; 6 Cancer Care Ontario, Toronto, Ontario, Canada; 7 Centre for Addiction and Mental Health, Toronto, Ontario, Canada

**Keywords:** Ovarian cancer, Exercise, Cognitive behavioral therapy, Chemotherapy, Health-related quality of life, Epithelial ovarian cancer

## Abstract

**Background:**

Ovarian cancer has the highest mortality rate of all gynaecologic cancers. Faced with poor prognoses, stressful treatment effects and a high likelihood of recurrence, survivors must confront significant physical and psychological morbidities that negatively impact health-related quality of life. Frequently reported side effects include cancer-related fatigue, peripheral neuropathy, and psychological distress. Exercise and cognitive behavioral therapy interventions have counteracted such adverse effects in other cancer populations.

**Objective:**

To investigate the feasibility and benefits of a 24-week home-based exercise intervention, coordinated with 12 weeks of cognitive behavioral therapy (two sessions per month), developed for two types of patients diagnosed with epithelial ovarian cancer: 1) those undergoing primary treatment with adjuvant chemotherapy after primary surgery; 2) those on surveillance after completing treatment within the last 2 years.

**Methods:**

Participants were recruited from the Gynaecologic Oncology Clinic. Eligible participants completed baseline assessments and were provided with home-based exercise equipment. Cognitive behavioral therapy was provided every other week for patients via telephone. Assessments were completed at baseline (T1), 3 months (T2) and 6 months (T3).

**Results:**

19 of the 46 eligible patients approached were enrolled, with 7 patients in the treatment group and 12 in the surveillance group. There was a significant within group increase in peak VO_2_ from baseline to 6 months: F_(2,16)_ = 5.531, p = 0.015, partial η^2^ = 0.409.

**Conclusion:**

The combined 6-month exercise-cognitive behavioral therapy intervention was associated with significant increases in aerobic fitness in epithelial ovarian cancer patients assessed. These improvements were similar regardless of whether the patient was receiving chemotherapy or under surveillance.

## Introduction

Ovarian cancer is the 5th most common female cancer [1] and has the highest mortality of all gynaecologic cancers [2], with epithelial ovarian cancer (EOC) being the most prevalent subtype (accounting for ~90% of ovarian cancers) [3]. Most cases are diagnosed during advanced stages because of non-specific symptoms and the absence of effective early detection [3], with standard care typically consisting of cytoreductive surgery followed by platinum and taxane-based chemotherapy [4,5]. Common side effects include nausea, poor sleep, vomiting, lost appetite, alopecia, anemia, increased infection risk, peripheral neuropathy and cancer-related fatigue (CRF) [5,6]. These effects, in combination with the poor prognosis, contribute to depression, anxiety [7-10] and/or posttraumatic stress disorder (PTSD) symptoms [8]. For many years, ovarian cancer patients were viewed as a fragile, inactive cohort, with little attention directed towards physical activity interventions. Indeed, interventions have been largely aimed at improving ovarian cancer survivorship via drug treatment [4-6,11,12]. Nonetheless, current research indicate that ovarian cancer survivors who meet healthy physical activity guidelines self-report less CRF and better sleep, psychosocial functioning and HRQOL [13-15]. Psychological interventions have also been found to decrease CRF symptoms [16,17] anxiety, depression, and treatment side effects in cancer patients [18-32] with positive results extending to 4 months [27,28], 6 months [29], and 12 months post treatment [30,31], especially when interventions focus on better self-management and decision-making [33]. A study conducted with gynaecological cancer patients indicated that women perceive physical activity participation as important and beneficial in terms of improved psychological well-being and physical functioning [34]; hence when ovarian cancer patients were asked if they would participate in a physical activity program, 54% answered ‘yes’ and 33% answered “maybe” [15], with the majority of positive responders (69%) preferring interventions within 1 year of treatment completion and the remainder (31%) preferring to start during treatment [15]. Similarly, a randomized control trial assessing feasibility and efficacy of a physical activity behavioural change intervention in managing fatigue with gynaecological cancer survivors found that physical activity interventions for gynaecological cancer survivors is not only feasible in terms of participants’ programme adherence and evaluation but also with their improvement of reported fatigue [35].

As a result, based on data indicating exercise interventions and cognitive behavioral therapy (CBT) interventions counteract adversive effects in other cancer populations [36-46], specifically stimulating positive cognitive and cardiovascular responses that improve mood, sleep, physical functioning and reduce CRF [42], we created and piloted a combined intervention specifically for EOC patients. Such interventions are warranted as higher levels of physical inactivity, depression and anxiety are seen in ovarian cancer patients when compared to patients with other life threatening illnesses and the general population [7,9,10]. Studies found that interventions may specifically prevent the development or exacerbation of PTSD symptoms. One particular study conducted using psychometric analysis with ovarian cancer patients found that 14% of newly diagnosed patients qualified for sub-syndromal PTSD diagnoses [8].

While it is unexplored to combine a counseling intervention like CBT with a home-based exercise intervention, we wanted to gain the advantage of exercise-related reductions in CRF [36-39,41]. Studies by Pinto et al. and by Mock et al., found that home-based physical activity interventions had positive effects on HRQOL, fatigue levels and body composition for breast cancer patients [47,48], especially when coupled with brief telephone contact [47]. Additionally, the detriments of HRQOL associated with CRF have been reduced by exercise interventions in multiple cancer populations [36-39,41] leading to a consensual judgment that exercise can be an effective modality for reducing CRF [42,43]. Exercise alleviates CRF symptoms through adaptive cardiovascular responses and improvements in sleep quality and mood [42], advancing views that exercise, during cancer treatment, can generically reduce side-effects. Although it may seem counterintuitive that exercise alleviates CRF, findings have conversely suggested that prolonged bed rest and decreased activity lead to muscle mass loss and reduced cardiac output which, in turn, leads to decreased ability to perform daily tasks [42]. Interventions for breast cancer patients during and after chemotherapy and a previous study with ovarian cancer patients found that physically active patients reported reduced CRF and improved HRQOL, cardiorespiratory fitness, and physical functioning [13,49].

When psychological interventions are analyzed we find that a meta-analysis of 45 psychological intervention studies with cancer patients concluded all interventions were better than usual care in positively affecting patient’s psychosocial wellbeing McCorkle et al. found that for post-operative gynaecological cancer patients (61.8% diagnosed with primary ovarian cancer) [33], interventions aimed at better self-management and more active decision-making [33] were associated with less uncertainty and symptom distress, and improvements in mental/physical HRQOL [33]. Hence, similar to the exercise component, the CBT was designed to be home-based, delivered via the telephone given past indications of effectiveness [50-56].

This pilot study aimed to investigate the potential feasibility and benefits of an exercise-based intervention, coordinated with CBT, customized for invasive EOC patients who were: 1) undergoing primary treatment with adjuvant chemotherapy after primary surgery; or 2) on active surveillance after completing treatment within the last two years. One study objective, for each group, was identifying the stage of cancer care associated with the most feasible, effective and beneficial interventions combining exercise and CBT.

## Methods

This study was approved by the Research and Ethics Board at the University Health Network. Participants were recruited from the gynaecologic oncology outpatient clinics at the Princess Margaret Hospital (PMH)/University Health Network in Toronto, Ontario. Participants were recruited (treatment patients and patients on surveillance after treatment completion) with the general inclusion criteria being: 1) fluency in English; 2) absence of diagnosed psychosis, dementia, significant cardiovascular impairments (i.e. congestive heart failure, coronary artery disease, or uncontrolled hypertension) or disease recurrence; and 3) no contraindications to exercise. Participants were further stratified into two groups based on the following criteria: 1) participants eligible for the treatment phase group were newly diagnosed with EOC, fallopian or primary peritoneal cancer and had received no more than two cycles of platinum-based chemotherapy, had been treated with primary surgery and were asymptomatic disease, if metastatic disease was persistent after surgery; 2) Participants eligible for the surveillance phase group were women who completed treatment for ovarian, fallopian or primary peritoneal cancer but were no more than two years post-treatment, had no evidence of recurrent disease by physical examination, biochemistry and/or imaging, and currently were not receiving active treatment. Clinical information obtained from medical records included disease stage, treatment, past history of depression/anxiety, past history of hypertension/cardiac disease and functional status.

### Recruitment

Clinic lists were reviewed for eligibility by clinic physicians and patients were approached by the research coordinator based on eligibility. Patients in the treatment phase group were approached after surgery but before completing two cycles of adjuvant chemotherapy and the surveillance phase group were approached when they were transitioned to surveillance near the end of their treatment.

### Procedures

Eligible participants returned to the hospital for baseline assessments where they completed a questionnaire package consisting of a demographic profile, physical activity questionnaire and psychological functional and various health-related quality of life (HRQOL) questionnaires including anxiety, depression, self-efficacy, neuropathy, and fatigue. Self-report assessments were followed by physical assessments that included an aerobic fitness test. Baseline assessments were followed by identical assessments conducted at 12 weeks and 24 weeks.

At baseline assessment, participants were introduced to the home-based exercise training and provided individualized prescriptions and equipment: stability ball, yoga mat, and resistance bands (light, moderate and advanced tension levels). Participants received an individualized program designed by a certified exercise physiologist (CEP) to improve musculoskeletal and cardiovascular fitness. Each exercise prescription was individually structured based on light-moderate aerobic exercise (brisk walking) and 10 resistance training exercises: 1) stability ball wall squats; 2) push-ups (wall, modified or traditional); 3) resistance band seated row; 4) hamstring curl; 5) lateral raises; 6) triceps extension; 7) biceps curls; 8) upright rows; 9) stability ball crunches; 10) hip extension. Each exercise was initially performed for 2 sets of 8 to 12 repetitions, with increased sets and repetitions contingent on CEP judgment and guidance. The aerobic exercise component was paced at a moderate intensity, requiring 60–70% of the participant’s estimated heart rate maximum (from baseline assessment) or 4–7 on the Ratings of Perceived Exertion (RPE) on a 10 point scale. Physical activity was encouraged for 3 to 5 times per week for 30–60 min per session, progressively increasing RPE over 6 months. Participants completed aerobic and resistance trainings on alternate days and recorded weekly activity in a detailed exercise manual with exercise descriptions conveyed verbally and pictorially in coordination with step-by-step exercise safety guidelines.

CBT counselling sessions were completed by phone (between participant and CBT counsellor) for one hour duration every two weeks. The CBT intervention addressed topics such as death, recurrence, fear, anxiety, pain, hope, and happiness.

### Measures

#### Psychological and demographic questionnaire measures

Demographic data collected included: age, ethnicity, marital status, household income, education, history of psychological impairments, and history of physiological impairments. Other data collected include patient’s hospital records: age at diagnosis and stage of disease. Patient’s hospital records were used in addition to a questionnaire to gather relevant information. Self-reported HRQOL and Psychosocial Outcomes questionnaires were used: The Functional Assessment of Cancer Treatment-Ovary (FACT-O)which assessed ovarian related quality of life factors when combined with the FACT-G (FACT-general); FACIT-Fatigue: used to assess fatigue in cancer populations; FACT-ES: used to assess the effects of endocrine treatment; Profile of Mood States-SF Vigor Scale (POMS-SF-V): used to assess vigor and mood; FACT-GOG/NTX: an additional treatment-specific subscale used to assess chemotherapy-induced peripheral neuropathy; Center for Epidemiologic Studies-Depression Scales (CES-D): used to assess clinical / non-clinical levels of depression in clinical and community samples; State Trait Anxiety Inventory (STAI-Y): used to assess the current level of anxiety for both state and trait features; Posttraumatic Stress Symptoms*:* The Posttraumatic Stress Disorder Checklist – Civilian Version (PCL-C): used to assess post-traumatic stress symptomology; Cancer Behavior Inventory-Brief (CBI-B): used to assess cancer-coping self-efficacy; and Godin Leisure-Time Exercise Questionnaire-Leisure Score Index: used to assess exercise intensity and frequency.

#### Fitness measurements

Tests focused on cardiovascular status (resting heart rate and blood pressure), body composition (height(m), weight(kg), waist circumference (cm), and body fat percentage using skin folds), aerobic capacity (ml/kg/min) and muscular strength (grip dynamometry (kg)). Body fat was determined by the sum of three skinfolds using a skinfold caliper (triceps, suprailiac and the thigh) [57]. The Modified Bruce Protocol Treadmill Test was used to assess patients’ aerobic capacity, derived through a VO_2_ peak score. Participants were asked to walk to their maximum and the final speed and grade was converted into a VO_2_ peak score, according to ACSM’s metabolic equation [58]. Height (m) and weight (kg) were converted into a BMI score (kg/m^2^).

#### Statistical analysis

Statistical analysis was conducted using the Statistical Package for the Social Sciences version 17 [59]. Group differences in demographic characteristics were analyzed using chi square tests for categorical variables and independent t-tests for continuous variables. Following per protocol, repeated measures ANOVA was used to examine change over time for each variable across three time points (baseline, 3 months, and 6 months).

## Results

### Recruitment results

**Figure 1:**
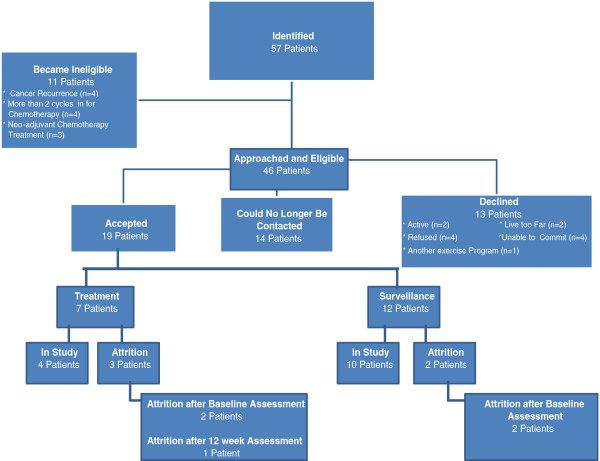
**Timeline of recruitment and involvement for treatment and surveillance participants.** Timeline of patients who were identified, recruited, declined, loss to follow-up and those who participated.

Patients were recruited over a period of four months (March 2011 to July 2011). Based on eligibility criteria, 57 patients were identified (Figure 1). During the time period given for potential participants to consider participation in the study, 11 patients were not eligible for the study.

The potential participants for the surveillance phase group were deemed not eligible due to cancer recurrence (n = 4); potential treatment phase participants were not eligible because they had received more than 2 cycles of chemotherapy (n = 4) or they were treated with neoadjuvant chemotherapy and did not receive primary cytoreductive surgery (n = 3).

Consequently of the 46 participants who were eligible, 13 patients declined and 14 could not be reached for follow-up contact. Reasons for participants declining were: felt they were already too active (n = 2), felt unable to commit (n = 4), lived too far away (n = 2), involved in another exercise program (n = 1) or refused to participate without reason (n = 4). A total of 19 patients agreed to participate in the study, yielding a 41% accrual rate, with 7 patients eligible for the treatment group, while 12 patients were eligible for the surveillance group. From the participants who agreed to participate there was a total attrition rate of approximately 26% (3 in treatment group, 2 surveillance group). In the treatment group, attrition was attributed to 2 drop-outs and 1 loss to follow-up while in the surveillance group, attrition was attributed to 1 loss to follow-up and 1 disease recurrence.

Table 1 displays the demographic variables for the participants for each group in the study. Between the treatment and surveillance groups, minimal differences were found in regards to marital status, education, income and age. However, some differences were observed with regards to stage of disease and ethnicity. Though not significant, there were proportionally more women with early staged cancer were in the surveillance group than in the treatment group (*χ*^2^ (1,19) = 2.423, p = 0.120).

**Table 1:** Demographic variables for sample divided by treatment status

**Demographic measures**		**Treatment**	**Surveillance**	**P value**^**a**^
**Marital status (n = 19)**				
	Married/Cohabiting	5 (26%)	5 (26%)	0.210
	Single/Divorced/Widowed	2 (11%)	7 (37%)	
**Ethnicity (n = 19)**				
	Caucasian	7 (37%)	7 (37%)	0.047^*^
	Other ethnicity	0 (0%)	5 (26%)	
**Education (n = 18)**				
	Some university or less than	4 (21%)	7 (37%)	0.892
	University/College or more	3(15.5%)	4 (21%)	
	Missing		1 (5%)	
**Income (n = 17)**				
	$80,00 and over	4 (21%)	4 (21%)	0.279
	Under $80,000	3 (15.5%)	6 (32%)	
	Refused		2 (11%)	
**Stage (n = 19)**				
	Stage I/II	1 (5%)	6 (32%)	0.236
	Stage III	6 (32%)	6 (32%)	
**Age (y) (n = 19)**				
	Mean age (SD)	52.7 (12.1)	57.8 (12.0)	0.385

^*^ Asymp. Sig (2 sided), p < .05.

^a^ p values based on sample sizes specified in \demographic measures column.

The treatment group was entirely made up of white women while the surveillance group was nearly equally divided between white and other ethnicities (Table 1), *χ*^2^(1,19) = 3.958, p = 0.047.

#### Intervention results

Results indicate the amount of participants who were assessed for all three time point.

### VO_2_ peak analysis

An independent t-test showed no statistical significant differences between the two groups at baseline, t_(16)_ = .139, p = 0.891. There was no significant main effect of group: F_(2,14)_ = 1.407, p = .277, partial η2 = 0.167 (Table 2). There was however a significant increase in VO_2_ peak over time: F_(2,14)_ = 6.905, p = .008, partial η2 = 0.497.

**Table 2:** Physiological and fitness variables for participants assessed at each time-point

	**N**	**Baseline**	**12 weeks**	**24 weeks**	**P value**
VO_2(ml/kg/min)_ Peak					
Treatment	3	30.0 (8.0)	36.8 (4.8)	38.3 (2.9) *	0.008^b^
Surveillance	6	29.1 (7.7)	30.7 (6.9)	33.0 (3.2)*	
Waist circumference					
Treatment	3	89.7 (18.6)	91.5 (19.9)	95.0 (20.1)*	0.027^b^
Surveillance	10	92.2 (12.4)	92.5 (12.1)	92.8 (11.2)*	
Body Fat %					
Treatment	3	57.7 (7.5)	53.7 (7.3)	61.0 (6.6)†‡	0.022^c^
Surveillance	10	67.4 (7.6)	67.5 (6.0)	65.7 (4.1)	

Values are means (SD).

^*^significantly different from time 1 within group.

^†^significant between groups (p < 0.05).

^‡^significantly different from time 1 between groups.

^b^ within group (overtime).

^c^ group by time interaction.

### Waist circumference

There were no baseline differences t_(17)_ = −.287, p = 0.778, group by time interaction or main effect of group: F_(2,22)_ = 2.660, p = .092, partial η2 = 0.195. There was, however, an increase in participant’s waist circumference overtime: F_(2,22)_ = 4.257, p = .027, partial η2 = 0.279 (Table 2).

### Body fat percentage

At baseline there was no significant group difference, t_(17)_ = −.851, p = 0.406. There was a significant group by time effect for body fat %, F_(2,22)_ = 4.562, p = 0.022, partial η2 = 0.293. Treatment group had a significant increase in body fat % from month 3 to month 6 with a mean difference of 7.2%, p = .002 (Table 2).

Physiological measure of grip strength did not show any statistically significant change for the participants nor did BMI.

Psychological questionnaire measures used in the study did not yield statistically significant changes for trait anxiety, CBI, POMS, PCL, CESD, and the FACT questionnaires (Table 3). Trait anxiety showed non- statistically significant decreases along with POMS, CESD and PTSD and all indicating that by the end of the intervention trait anxiety, mood, PTSD and depression were decreasing. Non-significant increases were seen with the CBI, and non-significant increases were seen with the FACT questionnaires for surveillance; indicating that by study completion participants’ level of confidence and self-efficacy and HRQOL was increasing.

**Table 3:** Psychological variables for participants assessed at each time-point

	**N**	**Baseline**	**12 weeks**	**24 weeks**	**P value**^**b**^
State anxiety					0.359
Treatment	3	35.7 (14.0)	42.7 (10.0)	52.3 (24.4)	
Surveillance	5	38.8 (15.1)	40.6 (18.0)	43.2 (18.3)	
Trait anxiety					0.080
Treatment	3	37.3 (9.6)	42.7 (8.1)	32.7 (11.0)	
Surveillance	5	34.4 (15.0)	36.4 (20.8)	34.8 (16.1)	
FACT-G					0.605
Treatment	3	75.7 (12.7)	67.3 (9.7)	73.3 (18.6)	
Surveillance	10	81.9 (19.0)	83.2 (16.3)	83.2 (17.7)	
FACT-ES					0.346
Treatment	3	104.0 (43.8)	119.0(16.5)	124.6 (17.1)	
Surveillance	10	138.3(26.4)	138.3 (26.5)	140.9 (25.2)	
FACT-O					0.625
Treatment	3	105.3 (20.5)	94.9 (10.16)	103.9 (22.7)	
Surveillance	10	116.3 (22.8)	117.8 (22.4)	117.3 (22.8)	
FACT-F					0.693
Treatment	3	103.3 (23.7)	96.9 (11.4)	105.3(27.8)	
Surveillance	10	123.4 (28.9)	124.2 (26.9)	124.5 (27.3)	
FACT-GOG					0.401
Treatment	3	108.3 (21.1)	99.9 (6.4)	105.6 (27.1)	
Surveillance	10	119.5 (21.6)	117.0 (22.4)	119.2 (23.0)	
CBI					0.742
Treatment	3	95.7 (11.0)	101.7 (9.9)	101.7 (9.6)	
Surveillance	3	94.7 (16.0)	100.0 (10.6)	89.0 (14.0)	
CESD					0.797
Treatment	3	16.3 (12.5)	17 (7.2)	13.3 (15.3)	
Surveillance	5	10.2 (8.0)	6 (6.6)	9.0 (9.6)	
PCL					0.270
Treatment	3	32.7 (8.1)	37.7 (9.0)	29.0 (10.6)	
Surveillance	8	33.5 (10.2)	33.0 (11.0)	31.3 (11.8)	
POMS					0.983
Treatment	3	26 (3.5)	34.0 (14.0)	30.7 (7.5)	
Surveillance	10	41.4 (15.8)	35.1 (27.0)	39.4 (19.7)	

Values are means (SD).

^b^ within group (overtime).

Table 4 depicts the number of sessions that were completed for the CBT portion of the study. Although attrition did occur, CBT sessions were accounted for in the total of all participants as well as at study completion. Of the 19 participants, 5 did not complete all time point assessments. Of the remaining 14 participants who completed the intervention assessment time points, 13 of them completed 9 or more (over 75%) of the bi-weekly CBT telephone sessions. No statistical significance was found between the two groups of women, nor did they statistically affect the outcome variables.

**Table 4:** Completed cognitive behavioral therapy counselling sessions for participants

	**1/12 (%)**	**2/12 (%)**	**3/12 (%)**	**4/12 (%)**	**5/12 (%)**	**6/12 (%)**	**7/12 (%)**	**8/12 (%)**	**9/12 (%)**	**10/12 (%)**	**11/12 (%)**	**12/12 (%)**
Treatment	1 (5%)	1 (5%)							2 (11%)	1 (5%)		2 (11%)
Surveillance								1 (5%)	1 (5%)	3 (11%)	3 (16%)	3 (16%)
Total for all participants (N = 19)	1 (5%)	1 (5%)					1 (5%)	1 (5%)	3 (16%)	4 (21%)	3 (16%)	5 (26%)
Lost to attrition	1 (5%)	1 (5%)					1 (5%)			2 (11%)		
Total at study completion (n = 14)	0 (0%)	0 (0%)					0 (0%)	1 (5%)	3 (16%)	2 (11%)	3 (16%)	5 (26%)

## Discussion

There was a significant increase in aerobic fitness from baseline to 6 months in both treatment and surveillance patients. Although adherence to the exercise program was not available, the increase in VO_2_ peak suggests that participants likely participated in some form of regular physical activity which leads to improved cardiovascular functioning. A recent meta-analysis reports that aerobic exercise interventions improve cardiopulmonary function and body composition in women with breast cancer [60]. There were significant increases in waist circumference and body fat percentage that is typically seen with chemotherapy treatment [61]. The greatest weight gain is typically observed in women who become menopausal during treatment [61]. Participants with the greatest gain in body fat percentage may have gained weight as a result of treatment related effects. It can be attributed to treatment related changes such as hormonal changes, supportive medication given such as steroids to prevent nausea and psychosocial factors [61]. However, it is unclear whether the gain in adiposity was attenuated by exercise or whether the increased cardiovascular function resulted from the intervention given the absence of a control condition and adherence data.

Sample size was relatively small in this study, challenging the generalizability of findings. However, the large effect sizes still enable us to attain statistical significance in many outcomes and thus a larger sample size may not have been needed [62]. It is reasonable to take note of the non-significant changes in the data that could be clinically significant. For example, there were non-significant decreases in trait anxiety scale. In regards to the trait anxiety measure, recent studies have demonstrated trait anxiety changes can be attributed to the effects of interventions [63-65]. One interesting example is a frequently cited study by Davidson et al. where a mindfulness meditation training intervention, in a randomized controlled trial, resulted in significantly lower post-treatment trait anxiety scores when compared to randomly allocated wait-list controls [66], suggesting trait anxiety is a relevant outcome measure in behavioral interventions when an appropriate sample size can be recruited. Increasing CBI scores observed indicate higher levels of confidence at time 3 (vs. time 1) while POMS, PCL and CESD were decreasing indicating less symptomology of depression, post-traumatic stress disorder and mood disturbances at time 3 (vs. time 1). Results from the FACT-G, FACT-O, FACT-ES, FACT-F, and FACT-GOG/Ntx indicate very modest increases in HRQOL factors by 6 months for participants of the surveillance group. On average, women in the surveillance group reported higher HRQOL benefits than women in the treatment group. Predictably, during active treatment, HRQOL factors become more disrupted. Perhaps HRQOL increases the more removed the patient is from active treatment [67].

Participants in the study all completed more than half of the telephone based cognitive behavioural therapy counselling sessions. It was found that the CBT portion of the study provided no additional benefits, as everyone in the study adhered to the CBT counselling. Although no immediate benefits for CBT were observed, it is suggested that CBT interventions are most beneficial on QOL factors in longer-term follow-up periods [45,46]. Suggesting that in the future a post intervention assessment should be conducted to better understand the affect CBT has on QOL measures.

### Discussion of results overview

Statistical significance was found with a limited sample size, providing evidence that EOC patients are capable of participating in an exercise program especially when the exercise program employs a home-based approach, validating the evidence found by Stevinson et al. [15].

### Limitations and future direction

This study assessed psychological distress and quality of life findings through self-report questionnaires. With such a small sample size, the generalizability of these findings is limited. Another limitation of the study is the lack of fitness adherence data. We were unable to link each participant’s increase in VO_2_ peak directly to exercise program adherence although increased fitness levels are typically associated with increased exercise of some kind. Nonetheless, this was the first study to date to implement an exercise intervention with ovarian cancer patients. Due to the uncertainty of the cohorts’ physical fitness and overall capabilities, muscular strength was determined solely by grip strength. For a group of women who may experience neuropathy in their hands, this may not be an optimal assessment of true musculoskeletal fitness. In addition to the grip strength, assessments like the stand/sit and reach test for flexibility and the partial curl-up for abdominal and back fitness, might optimally be included in an overall evaluation of patients’ muscular fitness, according to the age appropriate guidelines for gender [58]. Despite the small sample size this pilot study brings awareness to a cohort of women who have shown interest in physical activity regardless of treatment difficulties and treatment trajectory [15].

This study was designed to assess whether an exercise-CBT intervention was feasible and beneficial for ovarian cancer patients. Although overall fitness benefits were observed in subjects, the pilot nature of the study precluded use of a non-intervention control group. Nonetheless, the study provided a platform for further studies which can more rigorously test the interventions. An important future direction is a randomized control trial, with adequate sample size. The overall goal is to provide ovarian cancer patients with better HRQOL after diagnosis, while bringing attention to the medical and health care system of the overall benefits that exercise and counseling can have during a patients’ treatment and coping trajectory post diagnosis.

## Conclusion

Results from this pilot study of a home-based exercise intervention coupled with cognitive behavioral intervention therapy sessions for ovarian cancer patients indicate significant increases in VO_2_ peak over time from baseline to 6 months. Focusing on patients’ HRQOL factors, increases from baseline to 6 months were seen in the FACT questionnaires amongst the participants, suggesting that regardless of what point in the trajectory of treatment a patient is at, some HRQOL benefits may be seen (in chemotherapy or on surveillance). Ultimately, this pilot study has provided evidence that an exercise intervention aimed at increasing cardiorespiratory fitness with ovarian cancer patients is possible and potentially beneficial, feasible and effective at two points in the ovarian cancer coping trajectory.

## Abbreviations

(BMI): Body Mass Index; (CBI-B): Cancer Behavior Inventory-Brief; (CRF): Cancer-Related Fatigue; (CES-D): Center for Epidemiologic Studies Depression Scale; (CBT): Cognitive Behavioral Therapy; (FACT-ES): Functional Assessment of Cancer Therapy-Endocrine Subscale; (FACIT-F): Functional Assessment of Chronic Illness Therapy-Fatigue; (FACT-G): Functional Assessment of Cancer Therapy-General; (FACT GOG-Ntx): Functional Assessment of Cancer Therapy/Gynecologic Oncology Group–Neurotoxicity; (FACT-O): Functional Assessment of Cancer Therapy-Ovarian; (FACIT): Functional Assessment of Chronic Illness Therapy; (HRQOL): Scales Health-Related Quality of Life; (PTSD): Posttraumatic Stress Disorder; (PCL_C): Posttraumatic Stress Disorder Check List Civilian Version; (POMS-SF): Profile of Mood States-Short Form; (STAI-Y): State Trait Anxiety Inventory

## Competing interests

The authors declare that they have no competing interests.

## Authors’ contributions

SM, CG, DS, SF, JK and PR conceived of the study, participated in its design and coordination and drafted the manuscript. SM and JK performed the statistical analysis. All authors read and approved the final version of the manuscript.
